# Safety and Effectiveness of the KA Micro Plug for Treatment of Patent Ductus Arteriosus in Preterm Low-Birth-Weight Infants: Retrospective Analysis From a Multicenter Registry

**DOI:** 10.1016/j.jscai.2026.105309

**Published:** 2026-04-09

**Authors:** Peter Guyon, Brent M. Gordon, Osamah Aldoss, Bassel Mohammad Nijres, Surendranath R. Veeram Reddy, Abhay Divekar, Thomas Forbes, Howaida El-Said

**Affiliations:** a Joe DiMaggio Children’s Hospital, Hollywood, Florida; b Rady Children’s Hospital, University of California San Diego, San Diego, California; c University of Iowa Health Care Stead Family Children’s Hospital, Iowa City, Iowa; d Children’s Medical Center Dallas, University of Texas Southwestern Medical Center, Dallas, Texas

**Keywords:** KA Micro Plug, low-birth-weight infant, patent ductus arteriosus, preterm infant, surgical ligation, transcatheter occlusion

## Abstract

**Background:**

Transcatheter occlusion of patent ductus arteriosus (PDA) in preterm infants is becoming a preferred approach when medical therapy fails or is contraindicated. This study was conducted to describe real-world clinical outcomes following transcatheter occlusion of PDA with the KA Micro Plug.

**Methods:**

This retrospective analysis was conducted using medical records from preterm infants (600-2500 g) with PDA treated with the Micro Plug Set (“KA Micro Plug”) across 4 US hospitals (January 1, 2020 to February 28, 2024). The primary safety end point was freedom from major adverse events through 30 days postprocedure; adverse events >30 days were also reported. The primary effectiveness end point was clinical success 6 months postprocedure (absent or trivial PDA shunt confirmed via transthoracic echocardiography).

**Results:**

One hundred sixteen patients were analyzed (median age at catheterization: 29.0 days). The primary safety end point was met in 116/116 patients (100%). Clinical success at 6 months was achieved in 70/71 patients (98.6%). Adverse events reported within 30 days of the procedure included pericardial effusion (n = 1), increased tricuspid regurgitation not requiring intervention (n = 2), and device embolization with successful retrieval (n = 1). Coarctation of the aorta (n = 1) was discovered at a 6-month follow-up echocardiogram. Three deaths occurred that were unrelated to the device or procedure.

**Conclusions:**

The KA Micro Plug demonstrated a high rate of clinical success and few adverse events. A prospective trial with the goal of approval by the Food and Drug Administration for use in this unique patient population is underway.

## Introduction

Preterm infants have a high incidence of patent ductus arteriosus (PDA).^1^ Clinically, PDA is associated with numerous life-threatening comorbidities, such as chronic respiratory disease, pulmonary hemorrhage, necrotizing enterocolitis, and renal insufficiency/failure.^2^ In addition to ongoing comorbidities, PDA is associated with prolonged hospitalization and an increased mortality risk.^3^^,^^4^

Despite the known risks associated with PDA, consensus regarding the need for closure is lacking. Currently, the decision for treatment of PDA is based on factors such as patient characteristics, echocardiogram parameters, and physician expertise/experience with available treatment options at a particular center. Management options are watchful waiting and/or pharmacologic closure (eg, cyclooxygenase inhibitors, paracetamol), surgical ligation, and, more recently, transcatheter occlusion.^2^ Surgical ligation was historically considered the standard treatment for definitive PDA closure in preterm infants when the condition was unresponsive to pharmacotherapy.^5^ Although surgical ligation is associated with high rates of clinical success, there are significant risks associated with the procedure, including vocal cord paralysis, hemorrhage, infection, and death.^6^^,^^7^

Transcatheter PDA (tcPDA) occlusion was developed more recently as an alternative to surgical ligation and quickly gained favor due to its less invasive nature and favorable morbidity profile. An emerging body of evidence has demonstrated that tcPDA occlusion is as effective as surgical ligation but carries a lower incidence of complications.^8^, ^9^, ^10^, ^11^, ^12^, ^13^, ^14^, ^15^ As a newer intervention, there is an ongoing need for evidence confirming the clinical benefit of tcPDA occlusion. The only device currently approved by the US Food and Drug Administration (FDA) for tcPDA occlusion in preterm infants is the Amplatzer Piccolo Occluder (Abbott)—all other devices are currently being used off-label. An investigational device exemption trial, the PREEMIE study,^16^ is underway to evaluate safety and efficacy outcomes of the Bloom Micro Occluder (formerly KA Micro Plug) for tcPDA occlusion in preterm infants. In this article, we offer a retrospective study describing real-world safety and effectiveness outcomes of tcPDA occlusion with the KA Micro Plug in preterm low-birth-weight infants across 4 different hospital systems in the United States.

## Methods

### Study design

This was a retrospective cohort study conducted across 4 US medical centers that are part of the Congenital Cardiovascular Interventional Study Consortium. All centers actively used the Micro Plug Set (“KA Micro Plug,” KA Medical/Merit Medical) for tcPDA occlusion between January 1, 2020, and February 28, 2024. Clinical data were obtained from the Congenital Cardiovascular Interventional Study Consortium database for patients who met the following inclusion criteria: (1) ≥5 days at time of treatment, (2) treated for PDA with the KA Micro Plug, (3) PDA minimum diameter was ≤4.5 mm and ≥3 mm in length, and (4) weight between 600 and 2500 g at time of treatment. Patients were excluded if they met any of the following criteria: (1) preexisting coarctation of the aorta, (2) preexisting left pulmonary artery stenosis, (3) cardiac output dependent on right to left shunt through the PDA due to supra systemic pulmonary hypertension, (4) intracardiac thrombus at the implant site or sign of venous thrombus in a vessel in which the device was inserted and delivered, (5) other known hemodynamically significant congenital heart disease at the time of treatment that required intervention, and (6) active infection.

### Ethical statement

All participating hospitals that contributed to this study received approval from their Institutional Review Board for data collected in this analysis. Informed consent was obtained from patients’ parents or a legally authorized representative.

### Device and procedure

**Figure 1:**
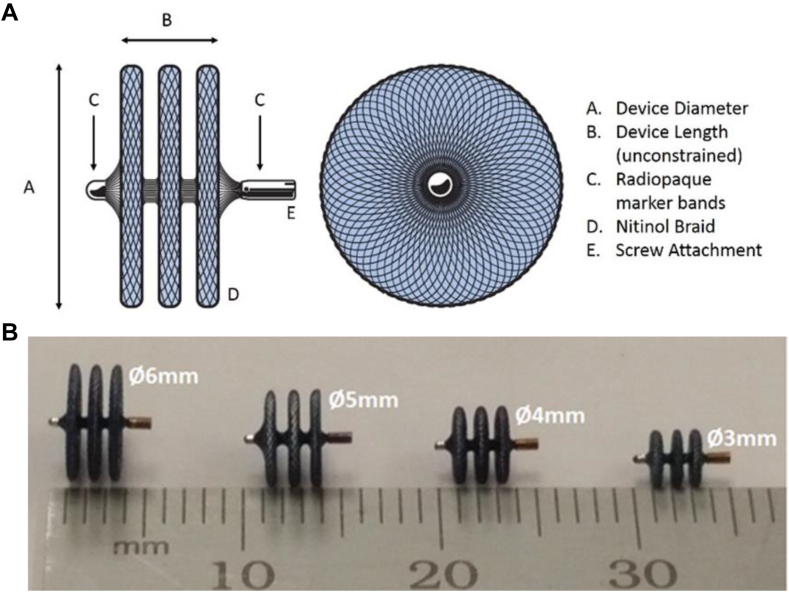
**KA Micro Plug (A) labeled illustration and (B) photo of available sizes.** Note that all 3 discs are of equal diameter. The unconstrained length (proximal disk to distal disk) is approximately 2.5 mm.

The KA Micro Plug system includes a micro occluder device and components used for implantation. The microoccluder is a soft self-expanding braided nitinol occlusion device available in 4 diameters (3, 4, 5, and 6 mm), with all 3 discs identical in diameter and an unconstrained length from proximal disk to distal disk of approximately 2.5 mm. The device is delivered through an included 2.9F microcatheter. The device has radiopaque marker bands attached to each end and a screw attachment for connection to a delivery wire (Figure 1, Central Illustration).

**Central Illustration:**
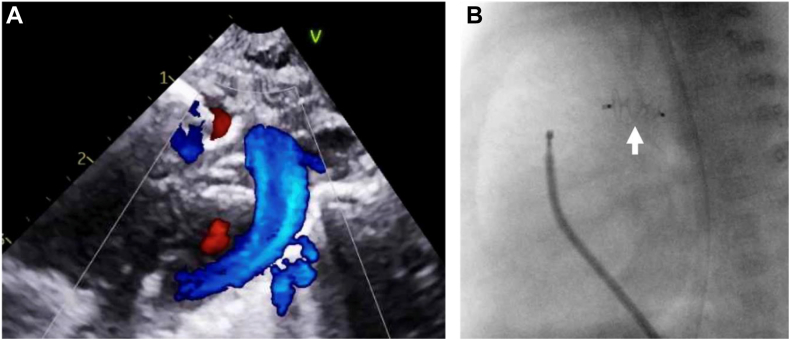
**Use of the KA Micro Plug in preterm low-birth-weight infants with PDA.** (**A**) Echo of the KA Micro Plug positioned within the PDA (highlighting its visibility on echo). (**B**) Angiographic image of the KA Micro Plug in position with the angled glide catheter and cable still within the main pulmonary artery before removal after releasing the device. The white arrow points to the device, highlighting fluoroscopic visibility.

TcPDA occlusion was performed either in the catheterization laboratory or bedside, following each hospital’s standard procedures. Under general anesthesia, transfemoral venous access enabled an antegrade approach. Initial transthoracic echocardiography confirmed the PDA’s presence and size. Using a combination of echocardiogram imaging and fluoroscopy, the right heart was traversed using a 4F Angle Glidecath catheter (Terumo Medical Corporation) and specific wires or microcatheters used at the discretion of the interventionalist. Angiography, device sizing, and deployment were performed as previously described,^11^^,^^15^ or as otherwise directed by the implanting physician.

### Study measures and outcomes

The primary safety end point was freedom from major adverse events (MAEs) through 30 days following device placement. The MAEs of interest consisted of the following: (1) device-related death, (2) conversion to open surgical repair related to device failure, and (3) hemodynamically significant adjacent vessel stenosis, such as left pulmonary artery stenosis or coarctation of the aorta that required further treatment (ie, surgical or transcatheter intervention). Significant left pulmonary stenosis was defined as a peak instantaneous gradient in the left pulmonary artery ≥35 mm Hg by echocardiogram if a lung perfusion scan was not available. For coarctation of the aorta, significant obstruction was defined as a gradient of ≥20 mm Hg in the aortic isthmus by invasive aortic catheterization or a mean gradient of ≥20 mm Hg in the aortic isthmus by echocardiogram if invasive aortic catheterization was not available. The presence of increased tricuspid valve regurgitation by 1 grade from baseline was also tracked and reported.

The primary effectiveness end point was the proportion of patients who achieved clinical success 6 months following placement of the device. Clinical success was defined as absent, or trivial residual PDA shunt (ie, grades 0 or 1), no significant left pulmonary artery stenosis or aortic arch obstruction, and no serious adverse device effects.

Adverse events were tracked and recorded during each case and following device placement until the point of the last follow-up echocardiogram.

### Statistical analysis

Descriptive statistics were used to summarize results. Continuous data were reported as median, interquartile range, and 95% CI. Categorical data were summarized as frequency, counts, and percentages.

All analyses were conducted using SAS version 9.4 (SAS Institute).

## Results

### Patient demographics

Demographics and clinical characteristics of patients who underwent tcPDA occlusion with the KA Micro Plug are outlined in Table 1. A total of 116 patients (65 female infants) were included with a median gestational age of 25.0 weeks and a median birth weight of 710 g. Most patients (93/116; 80.2%) underwent attempted pharmacologic closure for their PDA, with nearly half of all patients (45.8%, n = 38) having received 2 attempts at pharmacologic closure. The 2 most common indications for tcPDA occlusion were left heart enlargement (81%, n = 94) and ventilator dependence (63.8%, n = 74) (Table 1). The average follow-up was 6.5 months.

**Table 1:** Patient demographics and baseline characteristics

Characteristics	N = 116
Gestational age at birth, wk	25.0 [2.0]
Female sex	65/116 (56.0%)
Birth weight, g	710 [310.0]
History of pharmacotherapy for PDA closure	93/116 (80.2%)
No. of attempts at pharmacologic closure of PDA	
1	18/83 (21.7%)
2	38/83 (45.8%)
3	23/83 (27.7%)
>3	4/83 (4.8%)
Primary indication for PDA occlusion^a^	
Left heart enlargement	94 (81.0%)
Pulmonary hypertension	9 (7.8%)
Ventilator dependence	74 (63.8%)
Renal insufficiency	3 (2.6%)
Necrotizing enterocolitis	8 (6.9%)
Intraventricular hemorrhage	7 (6.0%)

Values are median [IQR], n/N (%), or n (%).

PDA, patent ductus arteriosus.

a Patients could have >1 indication for PDA closure.

### Procedural characteristics

TcPDA occlusion was predominantly performed in the catheterization laboratory (87.1%) as compared with the bedside (12.9%). Key procedural characteristics are summarized in Table 2. The median age and weight at the time of tcPDA occlusion were 29 days and 1093.5 g, respectively. The most common PDA morphology was type F (69.8%, n = 81) followed by type E (16.4%, n = 19). The median pulmonary artery end/narrowest diameter was 2.3 mm. The median PDA length was 9.5 mm. The median quantity of contrast used was 2.1 mL/kg, and the median fluoroscopy time was 7.0 minutes (Table 2).

**Table 2:** Procedural characteristics

Characteristics	N = 116
Age at catheterization, d	29.0 [20.0]
Median weight at time of procedure, g	1093.5 [465.0]
PDA morphology	
Type A (conical)	5 (4.3%)
Type B (window)	0 (0%)
Type C (tubular)	6 (5.2%)
Type D (complex)	3 (2.6%)
Type E (elongated)	19 (16.4%)
Type F (fetal type)	81 (69.8%)
Unknown	2 (1.7%)
Angiographic PDA measurements	
Aortic end, mm	3.6 [1.0]
Pulmonary artery end/narrowest diameter, mm	2.3 [1.0]
Length, mm	9.5 [2.6]
Total contrast volume, mL/kg	2.1 [1.8]
Total fluoroscopy time, min	7.0 [4.0]

Values are median [IQR] or n (%).

PDA, patent ductus arteriosus.

There was no residual shunting noted for 108 (93.1%) patients, whereas 8 (6.9%) had trivial residual shunting on echocardiography immediately following the procedure. All but 1 of these trivial residual shunts completely resolved on follow-up echocardiography. The most common device size implanted was 4 mm (38.8%, n = 45); other sizes used were 5 mm (36.2%, n = 42), 3 mm (17.2%, n = 20), and 6 mm (7.8%, n = 9). Eighteen patients (15.5%) required a different-sized device than initially selected, and 1 patient required 3 different-sized devices.

### Clinical outcomes

On the most recent follow-up imaging, 115 patients had no evidence of residual shunt, and 1 patient had a trivial residual shunt. One patient had a gradient that was ≥20 mm Hg in the aorta found at a 6-month follow-up visit, which was eventually corrected surgically (details below). No patient had significant left pulmonary artery stenosis on the immediate postprocedure echocardiogram or at the time of the most recent follow-up visit. Two (1.7%) patients had increased tricuspid regurgitation compared with baseline identified on the postprocedure echocardiogram. No patient had evidence of worsened pulmonary valve regurgitation compared with baseline.

A total of 101/115 patients had 3-month follow-up data; 71/113 had 6-month follow-up data. After that, there was a drop-off in data so that only 19/113 patients had follow-up data past 12 months. All 116 patients met the primary safety end point and were free from MAEs through the first 30 days postprocedure (100%; 95% CI, 96.9; 100%). A total of 70/71 patients met the clinical success end point at 6 months (98.6%; 95% CI, 96.9; 100%) (Table 3).

**Table 3:** Summary of safety and effectiveness outcomes

Clinical outcome^a^	Patients n/N^b^ (%)	95% CI^c^
Freedom from major adverse events through 30 d following transcatheter PDA closure	116/116 (100%)	96.9-100
Clinical success^d^ at ≥6-month follow-up visit	70/71 (98.6%)	92.4-100.

PDA, patent ductus arteriosus.

a Patients are counted only once within each category.

b Denominators include all patients who completed follow-up within the timeframe indicated.

c Confidence interval from the corresponding normal approximation.

d Clinical success was defined as absent or trivial residual PDA shunt, no significant left pulmonary artery stenosis or aortic arch obstruction, and no serious adverse device effects.

### Adverse events

A total of 8 (6.9%) adverse events were observed throughout the entire duration of the follow-up period. These events included 1 pericardial effusion, 1 device embolization, 2 cases of increased tricuspid regurgitation, 1 coarctation of the aorta, and 3 deaths unrelated to the procedure (Table 4).

**Table 4:** Adverse event summary

Adverse event	N = 116
Minor adverse events <30 d after occlusion	
Pericardial effusion	1 (0.9%)
Device embolization^a^	1 (0.9%)
Increased tricuspid regurgitation from baseline	2 (1.7%)
All-cause death^b^	3 (2.6%)
Adverse events >30 d after occlusion	
Coarctation of the aorta	1 (0.9%)

Values are n (%).

a Device embolization likely occurred due to the incorrect sizing of the device.

b All-cause death unrelated to the KA Micro Plug or transcatheter occlusion of the patent ductus arteriosus.

The pericardial effusion occurred intraprocedurally, presumably due to wire perforation of the right atrial appendage, before introduction of the device. The effusion was drained intraprocedurally, and the case was completed without recurrence and without any additional problems.

Device embolization occurred in 1 patient with a type F PDA who initially received a 5-mm device that was deployed and released. The device embolized almost immediately upon release, presumably due to incorrect size selection related to ductal spasm. The 5-mm device was retrieved with a snare without sequelae. The device was then upsized, and a 6-mm KA Micro Plug was deployed and released that resulted in good device position and no residual shunt. There was no increase in tricuspid or pulmonary valve regurgitation from baseline in that case.

One of the patients who experienced increased tricuspid regurgitation progressed from trivial (baseline) to mild on the immediate postprocedure echocardiogram. There were no clinical changes, and subsequent echocardiograms demonstrated no additional changes. No interventions were required. The second patient with increased tricuspid regurgitation progressed from mild (baseline) to moderate on the immediate postprocedure echocardiogram. The patient had no clinical changes, and no intervention was undertaken. The level of regurgitation remained moderate at the most recent follow-up visit.

One patient developed coarctation of the aorta after tcPDA occlusion. During the index procedure, a 5-mm device was initially placed in the PDA but not released due to observed left pulmonary artery narrowing. After the device was recaptured, it was downsized to a 4-mm device that was successfully deployed and released in the PDA (implantation was completely intraductal). Initial follow-up echocardiogram 48 hours after the procedure showed no residual shunt and no evidence of aortic arch or left pulmonary artery obstruction. The patient was lost to follow-up until 6 months following the index procedure, at which point an echocardiogram demonstrated a narrowing within the aortic arch with mild left ventricular hypertrophy and a mean gradient by echocardiogram >20 mm Hg. There was also an arm-to-leg blood pressure gradient of 18 mm Hg. Advanced imaging was performed, which demonstrated discrete coarctation at the region of the aortic ampulla adjacent to the device. After observation for a few months, the family elected for surgical device extraction and coarctation repair. Surgical removal was successful, and the patient continued to do well 16 months postprocedure (time of most recent follow-up).

The 3 deaths that occurred after tcPDA occlusion were unrelated to the device or procedure. The first death occurred in a male infant born at 25 6/7 weeks who had sustained a grade IV intraventricular hemorrhage, which was identified prior to the procedure. He underwent a successful tcPDA occlusion using a 4-mm device at 13 days of life without complication. He recovered from the procedure, but due to the poor neurologic prognosis and other significant medical comorbidities, the family elected to withdraw support. The second death occurred in a male infant born at 27 3/7 weeks. He underwent uncomplicated tcPDA occlusion at 14 days of life using a 5-mm KA Micro Plug. Approximately 1 month following the procedure, the patient developed necrotizing enterocolitis with pneumatosis on radiograph. Exploratory laparoscopy at the bedside led to small bowel resection and temporary abdominal wall closure (Silo). The patient’s condition continued to deteriorate; following extensive consultation, the mother elected to withdraw support. The third death occurred in a female born at a 31-week gestational age who was transferred from an outside hospital for evaluation and treatment of myelomeningocele and other comorbidities including hydrocephalus, Chiari II malformation with ventriculomegaly, severe respiratory distress, chronic lung disease, biliary atresia, elevated creatinine since birth, and bilirubinemia. She underwent uncomplicated tcPDA occlusion at 25 days of life. After much discussion, the family chose not to proceed with biliary atresia surgery due to concerns for her quality of life and she passed away.

## Discussion

Results from this real-world, retrospective, multicenter registry demonstrate that tcPDA occlusion with the KA Micro Plug is a safe and effective procedure. Specifically, results from this analysis indicate a clinical success rate of 99% and no MAEs within the 30-day period following transcatheter occlusion. Findings from this analysis compare favorably with published literature to date on tcPDA occlusion using other devices.^8^, ^9^, ^10^, ^11^, ^12^, ^13^, ^14^, ^15^ A study published in 2022 evaluated outcomes following tcPDA occlusion in 58 premature infants (mean weight, 1.4 kg) utilizing various devices including the Microvascular Plug (Medtronic) (n = 25), the Amplatzer Piccolo Occluder (Abbott) (n = 8), and the KA Micro Plug (n = 25).^12^ The procedure was successful in all 58 infants evaluated, and no significant procedural safety events were reported.^12^ A more recent multicenter study compared these 3 devices across 278 premature infants.^17^ In that study, procedural success was 98% across all devices, with a low rate of complications, none of which required reintervention. Results from a recent meta-analysis of 28 studies that included 373 infants ≤1.5 kg indicated an overall adverse event rate of 17.7% (MAE rate, 7.0%; minor adverse event rate, 10.7%).^18^

Available evidence to date indicates that tcPDA occlusion is safe and effective. Given the high vulnerability of this patient population, ongoing studies (eg, PREEMIE,^16^ PIVOTAL^19^) are needed to confirm the benefits of the procedure. This is important since certain events, such as late coarctation of the aorta, are not well-understood. Late or delayed coarctation of the aorta, which was observed in 1 patient in this analysis, has been observed across all tcPDA occlusive devices. Some have theorized that delayed coarctation results from completely intraductal placement of the device with no overhang into the pulmonary artery. In this paradigm, the device might become pushed toward and extruded into the aorta as the PDA continues to constrict (spontaneous closure typically proceeds from a pulmonary artery-to-aorta direction).^20^ Further study is needed in this respect. With this and other risks notwithstanding, a recent guideline opined that if patient characteristics are suitable and there is sufficient institutional expertise (ie, personnel, experience, volume of cases), then tcPDA occlusion may be considered.^21^

Beyond the decision of whether transcatheter closure for PDA is indicated, there is an additional choice about which device to employ. To date, 1 device, the Amplatzer Piccolo Occluder, is approved for tcPDA occlusion specifically in patients ≥700 g. Approval from the US FDA was based on findings from the investigational device exemption (IDE) study comprising 50 patients and the continued access protocol study comprising 150 patients.^13^ Results from the IDE and continued access protocol study underscore the safety and effectiveness of tcPDA occlusion using the device, with an implantation success rate of 99% in patients ≤2 kg. However, there were some important adverse events noted: 2 transfusions, 1 episode of significant hemolysis, 1 aortic obstruction, 5 embolizations, 2 postprocedure migrations, and 5 episodes of increased tricuspid regurgitation.^13^ Publicly available data from the FDA’s Manufacturer and User Facility Device Experience database on review of the Amplatzer Piccolo Occluder indicate that between January 1, 2019, and February 11, 2025, there have been 33 reports of aortic coarctation, 25 reports of left pulmonary artery stenosis/obstruction, and 7 reports of increased tricuspid regurgitation.^22^ Although it is not possible to determine a rate for these events (the total number of implanted devices is unknown, and underreporting is possible), awareness of the reports may be helpful as physicians consider options for transcatheter occlusion of PDA.

It is true that the use of the KA Micro Plug for PDA closure in premature infants constitutes “off-label” use. That said, using the device for this indication fits broadly within the designated use (arterial embolization); furthermore, this usage follows a long-standing pattern of off-label device use in pediatric interventional cardiology, where the lack of purpose-built devices creates a need for use of off-label devices until creation of a larger market for pediatric devices and/or until more of these devices can receive FDA approval after appropriate study. Use of the KA Micro Plug for PDA closure in premature infants predated approval of the Piccolo device at some centers, and some operators in the group continued to utilize it even after Piccolo’s FDA approval, given some concerns about the Piccolo device and some perceived advantages of the KA device.^11^^,^^12^^,^^15^

First, many users of the KA Micro Plug report qualitatively increased “softness” or malleability when compared with the Piccolo device. Although empirical evidence is lacking on whether the KA Micro Plug is meaningfully “softer” than other devices, some of the authors hypothesize that the perceived softness could contribute to improved device positioning within the PDA after deployment.

Second, the KA Micro Plug delivery microcatheter is inserted coaxially through the original 4F catheter (Terumo Glidecath), requiring passage through the tricuspid and pulmonary valves only once. In contrast, the Piccolo device is delivered through its own purpose-built 4F delivery catheter (TorqVue LP). As a result, placement of the Piccolo device requires removal of the soft 4F catheter (eg, Terumo Glidecath) used for initially crossing the PDA and insertion of its catheter, necessitating crossing the tricuspid valve again with the stiffer delivery catheter. The elimination of an extra catheter exchange is thought to potentially reduce the likelihood of tricuspid valve damage.

Finally, the flexibility of the Glidecath and the KA Micro Plug delivery cable—compared with the stiffer delivery system and catheter of the Amplatzer Piccolo Occluder—is thought to facilitate more precise device placement by minimizing distortion of the ductal anatomy during deployment and release, enabling the operator to naturally align the device with the native ductal orientation. The increased overall flexibility is also thought to contribute to hemodynamic stability during placement by causing less distortion or “tenting” of the tricuspid valve. Admittedly, it is difficult to quantify or objectively measure these perceived differences, but we highlight them since they are opinions common to all the operators in the study.

These and other perceived advantages of the KA Micro Plug have been highlighted previously.^15^ Even if there is no true advantage of 1 device over the other, the availability of an additional device with a good safety and efficacy profile is expected to have an overall positive impact in this market, potentially providing more options for individual operators to choose from as they assess patients, particularly low-birth-weight infants who require the smallest, shortest, and most flexible device feasible for procedures. An IDE trial (PREEMIE)^16^ is underway to evaluate clinical outcomes of the Bloom Micro Occluder (formerly KA Micro Plug) with a shortened microcatheter. We anticipate that the shortened working length of 87-cm for the microcatheter will be optimal for use in the preterm low-birth-weight-infants undergoing tcPDA occlusion relative to the 125-cm catheter length included in the KA Micro Plug. This is important since most operators modify the microcatheter length in the KA Micro Plug kit. The shortened length evaluated in the PREEMIE study is expected to further streamline the procedure.

Although this analysis adds to the growing body of evidence confirming the safety and efficacy of the KA Micro Plug, the findings from this study should be considered within the context of specific limitations. First, as a single-arm cohort study, the magnitude of the clinical benefits following tcPDA occlusion was not directly compared with an alternative treatment or intervention. Moreover, our retrospective analysis included only patients treated with the KA Micro Plug and did not specifically query instances where a KA Micro Plug device was planned but another device was used instead. Second, although data were available on all 116 patients 30 days following the procedure, by 6 months, a significant number of patients were lost to follow-up. As a result, evidence on the long-term safety of the device is lacking and this would be a fruitful avenue for future study. Finally, although the criteria for eligibility were as comprehensive as possible, the retrospective design of the study is an inherent limitation and subject to potential selection bias and confounding variables. Despite these limitations, this analysis provides useful information for interventionists treating this uniquely vulnerable patient population.

## Conclusion

Findings from this retrospective analysis indicate that tcPDA occlusion with the KA Micro Plug is safe and effective. The authors opine that the KA Micro Plug also carries specific advantages compared with other devices currently used for tcPDA occlusion. These results will be valuable for neonatologists and interventional cardiologists as they make clinical decisions on treating this important and vulnerable patient population. A pivotal study is currently underway to confirm the safety and efficacy of the device in preterm low-birth-weight infants.

## Declaration of competing interest

Peter Guyon is a consultant for Merit Medical. Brent M. Gordon is a consultant for Merit Medical. Proctor for WL Gore and Abbott. Osamah Aldoss is a proctor and consultant for Medtronic. Surendranath R. Veeram Reddy is a consultant for Medtronic and Tremedics, Inc. Abhay Divekar is a consultant for Exemplar Genetics, LLC (DBA Precigen Exemplar). Thomas Forbes is a consultant Abbott, Medtronic, Gore, Occlutech, AcuNav Medical, and Siemens Medical. Howaida El-Said is a consultant for Merit Medical and Starlight Medical. Bassel Mohammad Nijres reported no financial interests.
